# Acute Fasting Modulates Food-Seeking Behavior and Neural Signaling in the Piriform Cortex

**DOI:** 10.3390/nu14194156

**Published:** 2022-10-06

**Authors:** Fung-Yin Ngo, Huanhuan Li, Huiqi Zhang, Chun-Yue Geoffrey Lau

**Affiliations:** 1 Department of Neuroscience, City University of Hong Kong, Hong Kong, China; 2 Shenzhen Research Institute, City University of Hong Kong, Shenzhen 518057, China

**Keywords:** hunger, synapses, olfactory cortex, plasticity, foraging

## Abstract

It is well known that the state of hunger can modulate hormones and hypothalamic neural circuits to drive food-seeking behavior and consumption. However, the role the sensory cortex plays in regulating foraging is much less explored. Here, we investigated whether acute fasting in mice can alter an odor-guided foraging behavior and how it can alter neurons and synapses in the (olfactory) piriform cortex (PC). Acute hunger enhances the motivation of a mouse to search for food pellets and increases food intake. The foraging behavior strongly activates the PC, as revealed by c-Fos immunostaining. The activation of PC is accompanied by an increase in excitation–inhibition ratio of synaptic density. Fasting also enhances the phosphorylation of AMP kinase, a biochemical energy regulator. Taken together, our results uncover a new regulatory brain region and implicate the PC in controlling foraging behavior.

## 1. Introduction

Hunger is a physiological state that induces homeostatic feeding in order to gain energy. Decades of research have revealed a great deal on the hormonal and hypothalamic control of food intake and metabolism [1,2,3,4]. The neuropeptide Y (NPY)/agouti-related protein (AGRP)-expressing, pro-opiomelanocortin (POMC)-expressing and melanocortin receptor-expressing neurons in the arcuate nucleus of hypothalamus are central to food and appetite regulation [5,6]. Hunger activates the AGRP neurons and drives food intake [7,8,9,10]. Inhibition of POMC neurons, partly by AGRP projections, suppresses food intake [5,11,12]. While the hypothalamic control of hunger and feeding behavior is well documented, the role of chemosensory signals from the environment in food foraging behavior and subsequent intake is less clear. Increasing evidence suggests that sensory systems, particularly the olfactory structures, play important roles in modulating foraging behavior. Hypothalamic NPY was recently shown to mediate hunger-dependent attraction to food odors, but it is unclear whether this signal affects sensory processing [13]. Deletion of olfactory sensory neurons in obese mice induces weight loss and improves insulin resistance, suggesting the sense of smell can influence global metabolism [14]. Furthermore, endocannabinoids suppress neural activity of feedback axons originating from the olfactory cortex and enhance odor detection and food intake [15]. Altogether, these studies suggest that the metabolic state of an animal can exert an important influence on olfaction apart from feeding behavior. Many of these studies assume that the modulation takes place in the olfactory bulb (OB), but how hunger regulates circuits in the higher olfactory cortex that code for odor valence and association remains unexplored.

The anterior piriform cortex (APC) is a primary sensory cortex one synapse downstream of OB, yet exhibits properties that resemble both a sensory and association cortex [16]. The layer 2 (L2) of APC contains a dense region of principal neurons (semilunar, SL, and superficial pyramidal, SP) that form the major output of APC projecting to downstream areas, such as the entorhinal and orbitofrontal cortex and mediodorsal thalamus [17]. Our group and others have shown that SL and SP neurons form two distinct circuits for representing information in the APC [18,19,20,21,22]. Since the APC sends feedback projections to OB to modulate neural representation and learning [23,24], regulation of SL and SP neuronal output will significantly impact activity in APC as well as OB.

What is the contribution of the piriform cortex (PC) to food-seeking behavior? Little is known about how the PC contributes to foraging and food intake behavior in mammals. The spatial tuning of posterior PC neurons may assist with odor-place learning and memory [25]. A role for nutrient, specifically amino acids, sensing has been proposed for the PC [26,27], but this role is still being debated [28]. Sensory detection of food can rapidly modulate the activity of AGRP and POMC neurons in the hypothalamus, but it is unclear whether olfaction or other senses play a role in this regulation [29]. The state of hunger or activation of AGRP neurons can strongly modulate some of the food cue response in the insular cortex [30]. However, how neurons in PC are affected by hunger is unclear.

Here, we investigated the impact of acute fasting on mouse foraging behavior and neural activity in the APC. We used a combination of food-search task (FST), immunofluorescent staining for synaptic markers, and Western blot analysis of intracellular signaling to examine how the state of hunger can influence foraging behavior and APC activity.

## 2. Materials and Methods

### 2.1. Animals

Adult C57BL/6J mice between 8 and 10 weeks old and of both genders were used in this study. Mice were self-bred in Laboratory Animal Research Unit, City University of Hong Kong, housed in groups and maintained on standard laboratory chow ad libitum. Further, 24 h prior to the behavioral task, mice were deprived of food while maintaining the water supply or continued with ad libitum food.

### 2.2. Food-Search Task (FST)

Nine Falcon tubes (50 mL) with 1 cm hole drilled at mid-length and at the top were placed upside-down in a 500 mm × 500 mm × 500 mm white arena in 3 × 3 array of 150 mm apart under light. Each animal was allowed to habituate in the arena for 10 min. After all animals were habituated, a food pellet was randomly placed in one of the tubes and each animal was placed in the arena for exploration again. All habituations and tests were recorded by a camera from the top. The arena and the tubes were cleaned with 70% ethanol after each trial to eliminate social cues. A new piece of food pellet was also placed in one of the tubes randomly before the next trial. The latency of which the animal sniffed the food-pellet directly through the drilled holes was counted manually. The exploration time of each animal in the proximity of all tubes was determined by ToxTrac animal tracking software (Umeå University, Umeå, Sweden). The 50 mL Falcon tubes have a radius of 14 mm. A circle with 22 mm radius was constructed around each tube in the tracking software (8 mm excess radius outside the tube), which corresponded to the length from nose to centroid of the animals that indicate close interaction with the tubes. The ratio of exploration time around the food-containing tube and the total exploration time was expressed in percentage.

To study the effects of familiar odor on the food foraging activity, an almond was placed in a different tube from the food pellet. After exploring in the arena for 10 min, mice were fasted for 24 h and their foraging behavior was tested again. The positions of almond and food were randomly allocated in all trials. The food recognition index in sated and fasted states was defined by the ratio of the difference in exploration time between food and almond and the sum of the two values ([t_food_ − t_almond_]/[t_food_ + t_almond_]).

To study the feeding behavior of animals in different states, each animal was placed in another chamber containing pre-weighed chow in a Petri dish for 30 min after FST. The content was weighed again after each trial and the difference in chow weight was calculated.

### 2.3. Immunofluorescent Staining

Mice were sacrificed immediately after behavior task or one hour afterwards in the case of c-Fos detection. Mice were deeply anesthetized with ketamine/xylazine mixture (100 mg/kg ketamine and 50 mg/kg xylazine), perfused with ice-cold 1X phosphate-buffered saline (PBS; pH 7.4; 4 °C), and fixed with 4% paraformaldehyde (PFA) in 1X PBS. The whole brain was post-fixed overnight in 4% PFA, followed by cryoprotection in 30% sucrose in PBS for 72 h at 4 °C. Brain sections of 50 µm thickness were cut with cryostat (HM525 NX, Thermo Fisher Scientific, Waltham, MA, USA). Sections were washed 3 × 5 min with PBS, followed by blocking in 10% normal goat serum in PBS. For detection of c-Fos, sections were double stained with rabbit anti-c-Fos and mouse anti-NeuN primary antibodies (1:500, Abcam, Cambridge, UK) overnight at 4 °C. For detection of synaptic markers, sections were stained by rabbit anti-VGlut1 (1:1000, Synaptic Systems, Göttingen, Germany) or mouse anti-GAD67 primary antibodies (1:1000, EMD Millipore, Burlington, MA, USA) overnight at 4 °C. Sections were washed 5 × 5 min with PBS at room temperature and incubated in Alexa Fluorochrome-conjugated anti-rabbit or anti-mouse secondary antibodies specific to each marker (1:1000, Jackson Immunoresearch, West Grove, PA, USA) and 4′,6-diamidino-2-phenylindole (DAPI, 1:10,000, Santa Cruz Biotechnology, Dallas, TX, USA) for 2 h at room temperature in darkness. After washing with PBS for 5 × 5 min, sections were mounted on slides in antifade mounting medium (Vector Laboratories, Newark, CA, USA) and stored in a dark box at 4 °C. Samples stained for c-Fos and NeuN were visualized by Nikon Eclipse Ni-E upright fluorescence microscope (Nikon, Tokyo, Japan), whereas VGlut1 or GAD67-stained samples were imaged by Zeiss LSM 880 Confocal Microscope with ZEN imaging software (Zeiss, Jena, Germany). Images of APC were acquired with a 4× or 40× objective.

### 2.4. Image Analysis

Images were imported to and analyzed in ImageJ (National Institutes of Health, Bethesda, MD, USA) as 8-bit images. The areas of c-Fos and NeuN immunoreactivities in L1, L2, and L3 of APC for different experimental conditions were quantified by the particle analyzing tool. The ratio between c-Fos and NeuN signals was calculated and expressed in percentage. For quantification of VGlut1+ or GAD67+ puncta density, a minimum threshold was chosen for each sublayer across experimental conditions to exclude background and include structure that appears punctate in ImageJ, while the maximal threshold was left at the highest value (255 for 8-bit images). The number of puncta and area of the image were determined by the particle analysis tool, which were expressed in ratio to obtain the puncta density per square millimeter.

### 2.5. Western Blotting

Animals were sacrificed by deep anesthesia of ketamine/xylazine mixture. APC was obtained by dissecting fresh brain tissue on ice and immediately snap frozen in dry ice. Samples were homogenized in radioimmunoprecipitation assay (RIPA) buffer supplemented with 1:200 protease inhibitor cocktail, 1 mM sodium pyrophosphate, and 20 mM sodium fluoride (all from Sigma-Aldrich, St. Louis, MO, USA), followed by centrifugation at 15,000 rpm for 15 min at 4 °C. Protein concentrations were measured by protein assay reagent (Bio-Rad, Hercules, CA, USA). A such, 30 µg of protein samples was resolved on a 10% sodium-dodecyl sulfate (SDS) polyacrylamide gel and the gel was transferred onto a polyvinylidene fluoride (PVDF) membrane (Bio-Rad, Hercules, CA, USA). The membrane was blocked by non-fat milk dissolved in 0.1% Tween-20 in Tris-buffered saline (TBS-T) for one hour at room temperature, then probed with primary antibodies specific to p-AMPK (T-172), AMPK, p-Akt (S-473), Akt, p-Erk1/2 (T202/Y204), Erk1/2 (all at 1:1000; all from Cell Signaling Tech. Inc, Danvers, MA, USA), and β-actin (1:5000; Cell Signaling Tech. Inc, Danvers, MA, USA) overnight at 4 °C. The membranes were washed in TBS-T briefly and incubated with HRP-conjugated anti-rabbit (1:2500; Sigma-Aldrich, St. Louis, MO, USA) or anti-mouse (1:5000; Sigma-Aldrich, St. Louis, MO, USA) secondary antibodies. After washing away excessive secondary antibodies with TBS-T, signals on the membranes were detected by enhanced chemiluminescence reagent (Thermo Fisher Scientific, Waltham, MA, USA) using the ChemiDoc imaging system (Bio-Rad, Hercules, CA, USA). Band intensities were quantified in ImageJ (NIH, Bethesda, MD, USA). Each band showed in the representative blot was from an individual mouse. The band intensity of each phosphorylated protein was normalized to that of the unphosphorylated state, then normalized to the sated group.

### 2.6. Statistical Analysis

Statistical analysis was performed using SPSS (IBM SPSS Statistics, Armonk, NY, USA). Extreme outliers were removed from the data based on the descriptive statistics, defined by values more extreme than first quartile (Q1) − 3 * inter-quartile range (IQR) or third quartile (Q3) + 3 * IQR. Mild outliers defined by values more extreme than Q1 − 1.5 * IQR or Q3 + 1.5 * IQR were removed if they were reasonable to be excluded and did not reduce sample size substantially. The normal distribution of each data set was checked by Shapiro–Wilk test. Data with normal distribution and equal variances were analyzed by one-way analysis of variance (ANOVA) and differences between three or more experimental conditions were checked by post hoc Bonferroni multiple comparison. Normally distributed data with only two experimental conditions were analyzed by Student’s *t*-test or Paired *t*-test. Data that did not pass tests for normality and equal variances were analyzed by independent sample Kruskal–Wallis test followed by Bonferroni multiple comparison or Mann–Whitney U test if only two experimental conditions were present. Data were presented in mean ± standard error of the mean (SEM). Thresholds for significance were indicated as * *p* < 0.05, ** *p* < 0.01, and *** *p* < 0.001. All figures were prepared in Prism 8 (GraphPad, San Diego, CA, USA), Excel (Microsoft Corporation, Redmond, WA, USA), and Illustrator CC (Adobe, San Jose, CA, USA).

## 3. Results

### 3.1. Acute Fasting Increases Food Search Behavior

**Figure 1:**
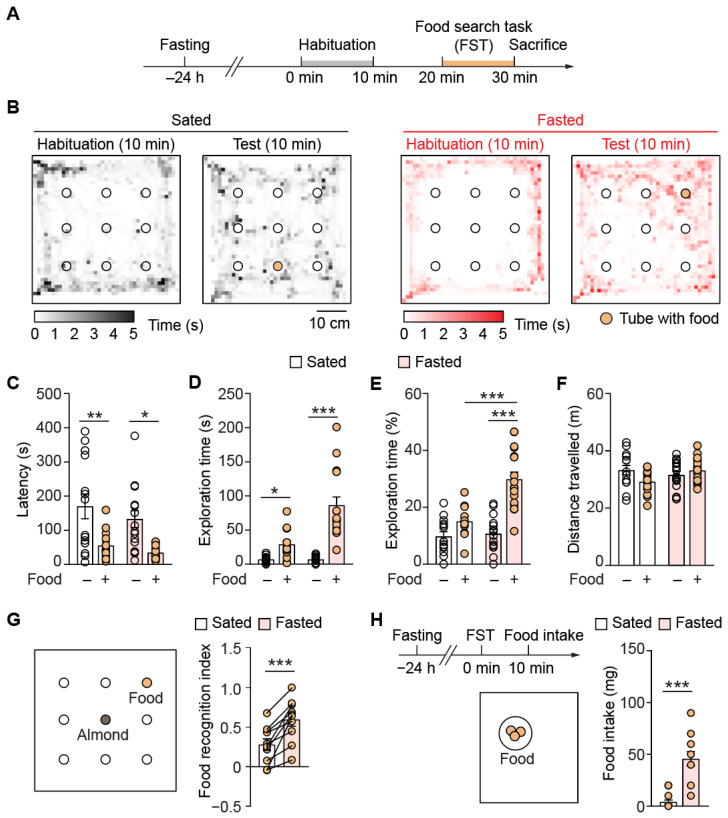
Acute fasting enhances food-seeking behavior. (**A**) Schematic of the food-search task (FST). Mice were first food deprived for 24 h, then subjected to 10 min of habituation in an arena with nine empty Falcon tubes. After 10 min, their food foraging behavior in the same arena was tested again, where one of them contained the target (food pellet). *n* = 14 and 17 for sated and fasted mice, respectively. (**B**) Heatmap showing the time spent in different regions of the arena during habituation or FST (representative experiment). Filled circle represents the food-containing tube. Mice spent little time foraging in the absence of food pellet but spent longer time exploring the tubes when food was added. (**C**) Quantification of the latency to explore the food-containing tube in FST, or the empty equivalent during habituation. Both sated and fasted animals had shorter latency to explore during FST compared to habituation. (**D**,**E**) Quantification of the exploration time of food (**D**) and the percentage to the total exploration time for all nine tubes (**E**). Both sated and fasted mice spent longer time in the proximity of the food in FST and fasted mice had a higher increase in percentage exploration time than sated mice in FST. (**F**) Distance travelled in the arena by sated and fasted mice during habituation and FST. (**G**) Design of the arena to study the difference in recognition of familiar food odor between sated and fasted mice and the quantification. An almond was placed in another tube apart from the food pellet. Exploration time to the two types of food was studied and food recognition index was calculated. After 24 h of fasting, the food recognition was tested again. Mice had higher recognition towards food pellet upon fasting (*n* = 10). (**H**) Timeline for measuring food consumed by animals and quantification. After FST, animals were placed in another chamber containing pre-weighed food on a Petri dish for 30 min for free intake of food. Fasted mice consumed more food than the sated mice (*n* = 16, 17 for sated and fasted groups, respectively). *, *p* < 0.05; **, *p* < 0.01; ***, *p* < 0.001.

To directly assess whether the state of hunger influences foraging behavior, we devised an odor-guided behavior task, the FST. Adult mice were divided into the sated (food accessible ad libitum) and fasted groups. The fasted mice lived in their original housing, but food chow was removed for 24 h while water was still available (Figure 1A). Mice were first habituated in the arena without any food pellet, then placed in the arena again with food chow in one of the tubes. Since each tube was sealed at the bottom, food odor could only escape through the two drilled holes at the higher positions and the animal could not eat the pellet. During the habituation phase, both sated (*n* = 14) and fasted mice (*n* = 17) spent little time examining the tubes. In contrast, both groups of animals explored around each tube for a longer time during the test phase that contained food in one of the tubes (Figure 1B). When food was present, both sated and fasted animals took less time to identify the same tube compared to habituation (sated-habituation to sated-FST: 170 ± 36 to 55.1 ± 11.9 s, *p* = 0.007, *n* = 14, 13; fasted-habituation to fasted-FST to: 133 ± 25 to 34.7 ± 4.6 s, *p* = 0.017, *n* = 16, 15; Figure 1C). The exploration time towards the food-containing tube also increased in both groups relative to their habituation phases, with the fasted group showing a significantly larger increase. However, the exploration time of fasted mice in FST did not differ significantly from that of sated mice in FST (sated-habituation to sated-FST: 7.0 ± 1.2 to 29.0 ± 5.9 s, *p* = 0.044, *n* = 14; fasted-habituation to fasted-FST: 6.9 ± 1.2 to 86.5 ± 11.9 s, *p* < 0.0001, *n* = 14, 17; sated-FST to fasted-FST: *p* = 0.059; Figure 1D). The fasted mice spent a significantly higher percentage of time exploring the food tube during the test phase (fasted-habituation to fasted-FST: 10.7 ± 1.5 to 29.8 ± 2.5 %, *p* < 0.001, *n* = 16, 17; Figure 1E), as well as the sated mice in FST (sated-FST to fasted FST: 15.0 ± 1.6 to 29.8 ± 2.5 %, *p* < 0.001, *n* = 12, 17; Figure 1E). Tracking of the animal in the arena revealed no difference in the distance travelled between habituation or test phase or between sated and fasted groups, suggesting that the locomotor activity of these mice was comparable (sated-habituation to sated-FST: 33.2 ± 1.6 to 29.2 ± 1.2 m, *p* = 0.225, *n* = 14, 13; fasted-habituation to fasted-FST: 31.5 ± 1.3 to 33.2 ± 1.0 m, *p* = 1.0, *n* = 16. 17; sated-FST to fasted-FST: *p* = 0.20; Figure 1F). To further test the mouse’s preference for different food-related odors, we placed an almond in another tube and compared the exploration time between the familiar food chow and almond odor. We computed the food recognition index, which was defined as (t_food_ − t_almond_)/(t_food_ + t_almond_). We found that fasted mice (*n* = 10) showed higher food recognition index than their own sated state, suggesting that the state of hunger increased the mice’s motivation to seek the food despite not being able to access it (sated to fasted: 0.3 ± 0.1 to 0.6 ± 0.1, *p* < 0.001, *n* = 10; Figure 1G).

Our previous results demonstrated that acutely fasted mice showed a higher tendency to attempt to procure food than sated mice, but do not inform whether they consumed more food. We next assessed food consumption by providing the mice with food pellets and allowed them to eat ad libitum following FST. Fasting greatly augmented the mice’s food consumption (*n* = 16, 17, respectively, for sated and fasted) (sated to fasted: 4.0 ± 1.9 mg to 45.7 ± 6.9 mg, *p* < 0.001, *n* = 15, 14; Figure 1H). These results correlated with the enhanced food seeking we observed (Figure 1C–G). Together, our results demonstrated that hunger enhanced foraging behavior and food intake and also established the effectiveness of FST in evaluating foraging behavior.

### 3.2. Foraging Activates the Anterior Piriform Cortex

**Figure 2:**
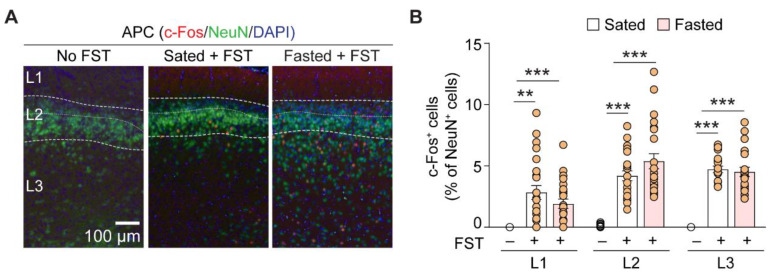
Foraging enhances neural activity in all layers of mouse anterior piriform cortex (APC). (**A**) Representative immunofluorescent images of the APC section from mouse that did not perform FST, sated and fasted mice that performed FST. The three layers of APC were outlined and annotated. (**B**) Ratio of c-Fos immunoreactive signal and NeuN immunoreactive signal across three APC layers among different experimental conditions, expressed in percentage. Mice that performed the FST showed marked activation of APC, as revealed by profuse expression of c-fos protein in various APC layers. However, the ratio did not differ significantly between sated and fasted mice across layers. *n* = 16–24 APC sections for each data set, obtained from 4–6 mice. **, *p* < 0.01; ***, *p* < 0.001. L1, layer 1; L2, layer 2; L3, layer 3.

Does searching for food involve sensory cortical areas, such as the APC? The APC is a three-layered paleocortex with cellular architecture that is similar to the hippocampus, with excitatory and inhibitory neurons arranged in specific layers. Layer 1 (L1) contains mostly neuropils and GABAergic interneurons. L2 contains a dense layer of principal neurons. Layer 3 (L3) contains sparsely distributed principal neuron somata and a variety of GABAergic interneurons. To address whether APC neurons are involved in food sensing and learning, we fixed and sectioned the APC for immunofluorescent staining of the immediate early gene, c-fos, after the mice underwent FST. Without FST, there was virtually no c-fos immunoreactivity in the APC across layers, suggesting the background neural activity was low (*n* = 16 APC sections, from 4 mice). After performing FST, the APC showed elevation in the ratio of c-fos-positive neurons in all three layers. However, there was no detectable difference in c-fos activation between sated and fasted mice (*n* = 24 APC sections from 6 mice for both groups; Figure 2A,B and Table 1). These results showed that following active searching for food chow, the sensory cortex is activated, presumably by the odorants emanating from it.

### 3.3. Acute Fasting Induces Plasticity of Excitatory and Inhibitory Synapses in the Anterior Piriform Cortex

**Figure 3:**
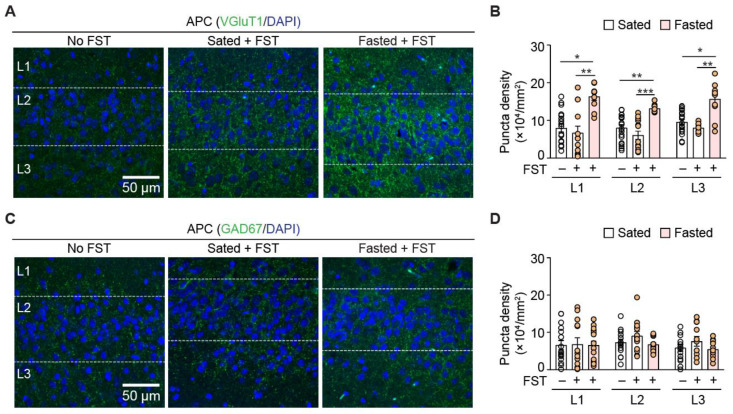
Acute fasting alters anatomical excitation at synapses. (**A**) Representative confocal images of excitatory presynaptic marker VGluT1 in APC for normal animals that did not perform FST, sated and fasted mice tested in FST. (**B**) Quantification of the density of VGluT1+ synaptic puncta in APC layers among different experimental conditions. Acute fasting increased VGluT1+ synaptic density compared to sated condition and normal mice. Density of VGluT1 did not differ between sated and normal mice, except a slight decrease in layer 3 APC of sated mice compared to normal mice. *n* = 13–19 APC sections obtained from 5 mice for each data set. (**C**) Representative confocal images of inhibitory presynaptic marker GAD67 in APC for animals that did not perform FST, sated and fasted mice tested in FST. (**D**) Quantification of the GAD67+ puncta density in APC layers among different experimental conditions. Acute fasting did not alter the puncta density across APC layers. *n* = 13–16 APC sections obtained from 5 mice for each data set. *, *p* < 0.05; **, *p* < 0.01; ***, *p* < 0.001.

What are the synaptic repercussions of FST-induced activation of APC? The complex interactions of excitatory and inhibitory local circuits are the driving forces of neuronal spiking activity. Activation of immediate-early genes, such as c-fos and associated transcription factors, can induce long-term synaptic plasticity. We hypothesized that acute fasting could alter the excitation–inhibition (E–I) balance in APC, thereby enhancing neural activity. We stained for a commonly used excitatory presynaptic marker, VGluT1, and a commonly used inhibitory presynaptic marker, GAD67. Prior to FST, the sated group showed similar density of VGluT1+ puncta in all layers to the normal mice. By contrast, FST in the fasted mice increased the density of VGluT1 synapses in all layers compared to sated mice with or without FST (*n* = 10 sections from 5 mice for all groups; Figure 3A,B, and Table 2). These results suggest that FST in the fasted mice can induce structural plasticity of excitatory synapses in the APC.

In contrast to excitatory synapses, acute fasting did not change the density of GAD67 synapses in all layers. The density of GAD67 synapses in all layers was also similar between sated mice with or without FST (*n* = 10 sections from 5 mice; Figure 3C,D and Table 3). Together, these results suggested that there was an overall increase in the excitatory synapses that changed the E–I balance in the APC following fasting.

### 3.4. Acute Fasting Enhances AMPK Phosphorylation and Decreases Akt Phosphorylation

**Figure 4:**
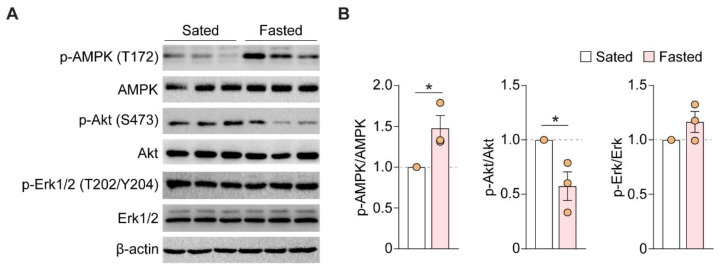
Acute fasting enhances p-AMPK and decreases p-Akt in APC. (**A**) Representative blot image for the expression of (p-)AMPK, (p-)Akt, (p-)Erk1/2, and β-actin. Thus, 30 μg of total protein from individual APC samples was used in each lane (*n* = 3 mice per group; each lane represents one individual mouse that performed FST). (**B**) Quantification of p-AMPK (T172)/AMPK (**left**), p-Akt (S473)/Akt (**middle**), and p-Erk1/2 (T202/T204)/Erk1/2 (**right**) ratio. Acute fasting enhanced phosphorylation of AMPK and decreased that of Akt compared to sated condition. *, *p* < 0.05. AMPK, AMP-activated protein kinase; p-AMPK, phospho-AMPK; Akt, protein kinase B; p-Akt, phospho-Akt; Erk1/2, extracellular signal-regulated kinase 1/2; p-Erk1/2, phospho-Erk1/2.

Does the fasting-induced enhancement in neural activity require specific signaling pathways? AMPK is a kinase that is linked to energy usage and homeostasis [31]. Activation of AMPK in *C. elegans* during hunger promotes foraging behaviors [32]. Moreover, activation of AMPK in the hypothalamus critically drives feeding in mice [33,34]. However, it is unclear whether AMPK and associated kinases in APC can be activated by feeding states. We tested whether acute fasting could alter phosphorylation of various kinases by performing Western blotting of APC samples from sated vs. fasted mice. Phosphorylation of AMPK at Thr172 in the α subunit is one of the best characterized activating mechanisms of this kinase [35]. Among the animals that performed FST, fasted mice showed increased phosphorylation of AMPK at Thr172 compared to sated mice, after normalizing to total AMPK (sated-FST to fasted-FST: 1.0 to 1.5 ± 0.2, *p* = 0.037, *n* = 3; Figure 4A,B). Akt can phosphorylate AMPK leading to its inhibition, whereas caloric restriction can reduce its inhibition on AMPK [35]. In the same samples, we found that acute fasting decreased phosphorylation of Akt at Ser143, normalized to total Akt (sated to fasted: 1.0 to 0.6 ± 0.1, *p* = 0.037, *n* = 3; Figure 4A,B). By contrast, fasting did not change ERK1/2 MAP kinase phosphorylation, normalized to total MAPK (sated to fasted: 1.0 to 1.2 ± 0.1, *p* = 0.487, *n* = 3; Figure 4A,B). These data corroborated with previous findings that activation of AMPK is often inversely correlated with Akt phosphorylation/activation. Altogether, these results showed that acute fasting can activate AMPK and alter phosphorylation signals specifically for each enzyme in the APC.

## 4. Discussion

The state of hunger can induce changes in hormonal status and neuronal circuitry in the brain to drive foraging behavior. Although much is known about how hypothalamic circuits can directly influence food procurement and intake, little is known about how sensory perception influences these behaviors [36]. Here, we show that acute fasting (24 h) enhances food searching behavior and activation of APC. This enhanced food exploratory activity is accompanied by changes in excitatory synaptic density and kinase activation in the APC. Altogether, these results suggest that neuronal and synaptic plasticity in the APC could enable enhanced foraging in the state of hunger.

**Figure 5:**
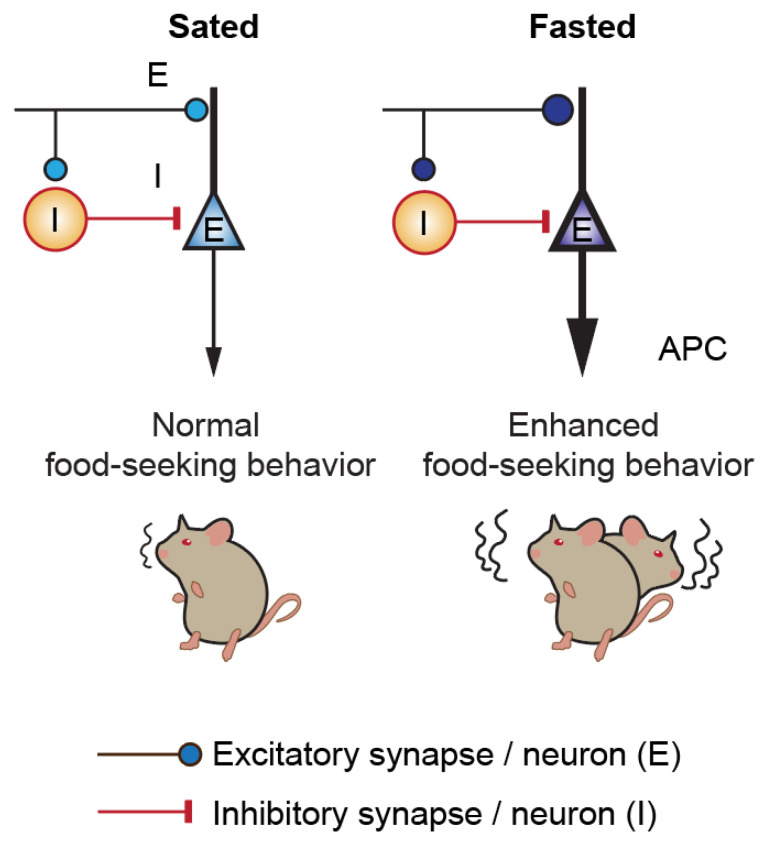
Acute fasting modulates excitation–inhibition balance in the APC and foraging behavior. Acute fasting enhances the excitatory synaptic expression, which could be an underlying mechanism in enhancing food-seeking behavior.

Hunger is a robust homeostatic state that induces negative valence signals in neural circuits to promote food search and consumption [37]. Although hunger is difficult to quantify, consummatory behavior, such as food intake, or appetitive behaviors, including food hoarding and food foraging, are relatively universal indicators of hunger among animal species [38]. Here, we show that acute fasting induces behavioral changes that are consistent with enhanced foraging and food intake behavior. We show that fasted animals exhibit shorter latency to locate the hidden food pellet, spend longer time in the proximity of food, and discriminate the food better than in the sated condition. This promotion of food seeking upon fasting is associated with APC and its plasticity, for instance, an increase in expression of an immediate-early gene, c-Fos, and an increase in excitatory synaptic density after fasting and FST. Most neurons in APC are principal neurons and they often have very low firing rates, both basally and upon odor stimulation [39]. This could be an explanation for the very low expression of c-fos before FST (Figure 1). Stimulus-transcription coupling, which involves activation of immediate-early genes upon stimulation, is conducive to long-term potentiation and depression in sensory processing [40]. After fasting and subsequent FST, we observed a significant increase in excitatory synaptic density, suggesting that structural plasticity of direct OB input and/or recurrent connections underwent long-term plastic changes. On the other hand, we did not observe a change in inhibitory synaptic density in APC after acute fasting, possibly because an increase in the presynaptic excitatory markers is a more rapid response than changing inhibitory expression (Figure 5). Although c-Fos activation is correlated to an increase in excitatory synaptic density, we do not know the causal mechanism. Two scenarios are possible: (1) c-fos activation leads to excitatory anatomical plasticity or (2) the enhanced excitatory synaptic density facilitates c-fos activation following FST. Further experiments are required to distinguish these scenarios. Western blotting shows that FST enhances phosphorylation and activation of AMPK in the fasted state. This is consistent with the literature, in that, in the energy-deplete state, AMPK is phosphorylated and activated [41]. As AMPK regulates diverse phenomena, such as glucose and lipid metabolism, inflammation and protein synthesis, it will be interesting to examine how fasting-induced activation of AMPK may affect these processes in APC. Although we identify APC activation following the behavioral task FST, we did not show that APC is causally linked to the enhanced foraging behavior. Future studies involving pharmacological, chemogenetic, or optogenetic manipulation of the APC will illustrate the functional role of APC in these state-dependent changes.

How can modulation of APC activity regulate appetite and foraging? The AGRP neuron is a major neuron type carrying the orexigenic signal in the hypothalamus and its activation alone could potently augment food intake [9]. Sensory stimuli, such as sight or smell, can rapidly reset the activity in AGRP and/or POMC neurons that leads to a decrease in hunger [29,37,42]. While it is increasingly clear how the neurons and circuits in the hypothalamus drive hunger-dependent food intake, much less is known about the nature and role of upstream sensory signals in modulating the feeding circuit. It is possible that olfactory information (learned or naïve) can arrive at the hypothalamus via multiple synaptic connections or changing the cognitive process that leads to a change in appetite. For instance, the outputs of APC relay to mediodorsal thalamus that is involved in cognitive flexibility may facilitate animals in choosing the best food reward [43]. In addition, hormonal signals, such as leptin, ghrelin and insulin, can modulate neural activity of various neurons in the hypothalamus [44]. Simultaneously, olfactory regions have been reported to express receptors for metabolic hormones and changed structurally with metabolic status [45,46]. It is possible that hormonal regulation of APC synapses and neuronal activity lead to activity changes in downstream brain regions, such as mediodorsal thalamus or insular cortex. More characterization studies on APC plasticity by hunger hormones are worth conducting to understand state-dependent changes in higher olfactory regions.

It has recently been shown that olfaction alone is not essential for high-fat diet-induced devaluation of standard diet, rather the consumption of high-fat diet [47]. In their study, mice fed with high-fat diet for a short period of time consumed less food chow, even when they were deprived of food overnight. Similar behaviors were observed in their anosmic animal model with ablated OB. Our results that hunger modulates neural activation and excitatory synapses in the APC are not in conflict with Boone et al.’s report, as they were only exposed to standard chow throughout the study and developed high familiarity towards it. Nevertheless, palatable food generally stimulates food-seeking and intake behavior. For instance, olfactory detection of peanut butter can enhance the long-term consumption of food chow [48]. Taken together, our study showed that acute fasting increases food foraging and intake in mice, accompanied by activation of APC that is important in odor perception and association. We believe that our findings will illuminate how biochemical and synaptic changes in APC can be involved in metabolic disorders and provide a basis for designing therapeutics for these disorders.

## Figures and Tables

**Table 1:** Expression of c-Fos across APC layers of sated and fasted mice with or without performing FST.

Layer	Contrast	c-fos^+^ Neurons (% of NeuN^+^ Cells)	*p*-Value	*n* (APC Section)
L1	Normal to sated	0 to 2.8 ± 0.6	<0.001	13, 24
	Normal to fasted	0 to 1.9 ± 0.4	0.001	13, 23
	Sated to fasted	2.8 ± 0.6 to 1.9 ± 0.4	1.0	24, 23
L2	Normal to sated	0.1 ± 0.04 to 4.2 ± 0.4	<0.001	13, 22
	Normal to fasted	0.1 ± 0.04 to 5.4 ± 0.6	<0.001	13, 23
	Sated to fasted	4.2 ± 0.4 to 5.4 ± 0.6	0.941	22, 23
L3	Normal to sated	0 to 4.7 ± 0.3	<0.001	13, 20
	Normal to fasted	0 to 4.5 ± 0.4	<0.001	13, 21
	Sated to fasted	4.7 ± 0.3 to 4.5 ± 0.4	1.0	20, 21

APC, anterior piriform cortex; FST, food-search task.

**Table 2:** The puncta density of VGluT1+ synapses across APC layers.

Layer	Contrast	Puncta Density (×10^4^/mm^2^)	*p*-Value	*n* (APC Section)
L1	Normal to sated	8.0 ± 1.0 to 6.7 ± 1.8	1.0	19, 13
	Normal to fasted	8.0 ± 1.0 to 16.3 ± 1.0	0.02	19, 10
	Sated to fasted	6.7 ± 1.8 to 16.3 ± 1.0	0.005	13, 10
L2	Normal to sated	8.0 ± 1.0 to 6.1 ± 1.1	0.617	19, 13
	Normal to fasted	8.0 ± 0.8 to 13.2 ± 0.3	0.009	19, 10
	Sated to fasted	6.1 ± 1.1 to 13.2 ± 0.3	<0.001	13, 10
L3	Normal to sated	9.6 ± 0.7 to 8.0 ± 0.4	0.757	19, 10
	Normal to fasted	9.6 ± 0.7 to 15.7 ± 1.5	0.011	19, 10
	Sated to fasted	8.0 ± 0.4 to 15.7 ± 1.5	0.001	10

**Table 3:** The puncta density of GAD67+ synapses across APC layers.

Layer	Contrast	Puncta Density (×10^4^/mm^2^)	*p*-Value	*n* (APC Section)
L1	Normal to sated	6.6 ± 1.2 to 6.8 ± 1.7	1.0	16, 13
	Normal to fasted	6.6 ± 1.2 to 6.6 ± 1.1	1.0	16
	Sated to fasted	6.8 ± 1.7 to 6.6 ± 1.1	1.0	13, 16
L2	Normal to sated	7.3 ± 0.9 to 9.1 ± 1.2	0.50	15, 13
	Normal to fasted	7.3 ± 0.9 to 6.8 ± 0.5	1.0	15, 16
	Sated to fasted	9.1 ± 1.2 to 6.8 ± 0.5	0.21	13, 16
L3	Normal to sated	5.9 ± 0.9 to 7.6 ± 1.3	0.67	15, 13
	Normal to fasted	5.9 ± 0.9 to 5.5 ± 0.6	1.0	15, 13
	Sated to fasted	7.6 ± 1.3 to 5.5 ± 0.6	1.0	13

## Data Availability

All data in this study are available from the corresponding author on reasonable request.
